# Risk Prediction Models for Patients with Head and Neck Cancer among the Taiwanese Population

**DOI:** 10.3390/cancers14215338

**Published:** 2022-10-29

**Authors:** Ming-Zhen Yu, Meei-Maan Wu, Huei-Tzu Chien, Chun-Ta Liao, Ming-Jang Su, Shiang-Fu Huang, Chih-Ching Yeh

**Affiliations:** 1 School of Public Health, College of Public Health, Taipei Medical University, Taipei City 110301, Taiwan; 2 Department of Public Health, School of Medicine, College of Medicine, Taipei Medical University, Taipei City 11031, Taiwan; 3 Master Program in Applied Epidemiology, College of Public Health, Taipei Medical University, Taipei City 110301, Taiwan; 4 Department of Nutrition and Health Sciences, Chang Gung University of Science and Technology, Taoyuan City 33303, Taiwan; 5 Research Center for Chinese Herbal Medicine, College of Human Ecology, Chang Gung University of Science and Technology, Taoyuan City 33303, Taiwan; 6 Department of Biotechnology, Ming Chuan University, Taoyuan City 33348, Taiwan; 7 Department of Otolaryngology & Head and Neck Surgery, Chang Gung Memorial Hospital, Taoyuan City 333, Taiwan; 8 Department of Laboratory Medicine, Shuang Ho Hospital, Taipei Medical University, New Taipei City 23561, Taiwan; 9 Graduate Institute of Clinical Medical Sciences, College of Medicine, Chang Gung University, Taoyuan City 333323, Taiwan; 10 Department of Public Health, College of Public Health, China Medical University, Taichung 406, Taiwan; 11 Cancer Center, Wan Fang Hospital, Taipei Medical University, Taipei City 116, Taiwan

**Keywords:** head and neck cancer, risk prediction models, sex difference, nomogram

## Abstract

**Simple Summary:**

Epidemiological evidence has suggested that modifiable factors play an essential role in the risk of head and neck cancer (HNC). However, few studies have established HNC prediction models based on sex and tumor subsites. In this study, we establish sex- and subsite-specific HNC risk prediction models for the general Taiwanese population. Our study draws from a large sample size of 14,423 participants. The HNC prediction models may be applied in clinical risk assessments for health promotion programs.

**Abstract:**

Epidemiological evidence has suggested that modifiable lifestyle factors play a significant role in the risk of head and neck cancer (HNC). However, few studies have established risk prediction models of HNC based on sex and tumor subsites. Therefore, we predicted HNC risk by creating a risk prediction model based on sex- and tumor subsites for the general Taiwanese population. This study adopted a case-control study design, including 2961 patients with HNC and 11,462 healthy controls. Multivariate logistic regression and nomograms were used to establish HNC risk prediction models, which were internally validated using bootstrap sampling. The multivariate logistic regression model indicated that age, education level, alcohol consumption, cigarette smoking, passive smoking, coffee consumption, and body mass index are common HNC predictors in both sexes, while the father’s ethnicity, betel-nut-chewing habits, and tea consumption were male-specific HNC predictors. The risk factors of the prediction model for the HNC tumor subsite among men were the same as those for all patients with HNC. Additionally, the risks of alcohol consumption, cigarette smoking, and betel nut chewing varied, based on the tumor subsite. A c-index ranging from 0.93 to 0.98 indicated that all prediction models had excellent predictive ability. We developed several HNC risk prediction models that may be useful in health promotion programs.

## 1. Introduction

Head and neck cancer (HNC) includes malignancies of the oral cavity, oropharynx, hypopharynx, and larynx [1]. Each year, over 900,000 HNC cases are diagnosed and 400,000 deaths from HNC occur worldwide [2]. In Taiwan, oral cavity (73.9%), oropharyngeal (11.2%), and hypopharyngeal (14.7%) cancers accounted for the majority of HNC in 2018. The incidence of HNC among men is higher than that among women. The age-adjusted incidence rate of oral cancer (including oropharyngeal and hypopharyngeal cancer) for men and women is 42.15 per 100,000 people and 3.92 per 100,000 people, respectively [3].

Epidemiological evidence suggests that modifiable lifestyle factors significantly affect the risk of HNC, but the associations vary, based on sex and tumor subsites. Studies have indicated that male smokers had a significantly greater risk of HNC than female smokers [4]. However, passive smoking and exposure to cooking oil fumes were reported to significantly increase the risk of oral cancer among Chinese women but not in men [5]. Alcohol consumption is another major risk factor for HNC, particularly for oropharyngeal and hypopharyngeal cancer [6]. Additionally, betel-nut chewing is a well-established risk factor for oral cancer, while combining tobacco consumption with betel-nut chewing is associated with a greater risk of oral cancer than oropharyngeal cancer [7].

Risk prediction models have been developed for many types of cancer, including breast, colorectal, prostate, lung, ovarian, and esophageal [8] cancers, but those for cancers in the head and neck are limited. Two studies in Japan have developed risk prediction models for upper aerodigestive tract cancer that included both genetic and environmental factors [9,10]. Hung et al. established a predictive model for oral cancer among high-risk individuals who had a habit of smoking or betel nut chewing in Taiwan [11]. As mentioned above, the risk of HNC varies by gender and tumor subsite. However, no study has established a risk prediction model of HNC that is based on sex and tumor subsites for a Taiwanese population. In the present study, we predicted the risk of HNC by using sex- and tumor subsite-specific models in the general Taiwanese population.

## 2. Materials and Methods

### 2.1. Study Participants

**Figure 1:**
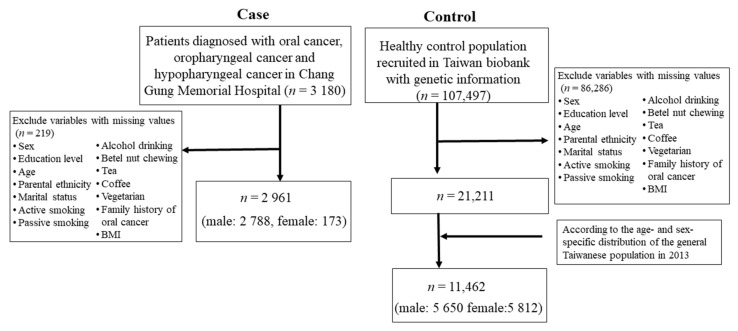
Selection procedure of participants with head and neck cancer.

We conducted a case-control study by combining a large series of HNC cases from a medical center in northern Taiwan with a large series of general noncancer controls of the Taiwanese population from the Taiwan Biobank (TBB). The TBB is an ongoing prospective cohort study, recruiting over 160,000 participants from the community across Taiwan since 2012 [12]. Eligible participants are aged over 30 years old and have no prior diagnosis of any cancer or human immunodeficiency virus infection. Upon their recruitment, the participants completed written informed consent, a demographic questionnaire, a physical examination, and laboratory blood and urine tests. A total of 3313 patients with HNC were recruited from the Department of Head and Neck Oncology at Linkou Chang Gung Memorial Hospital between 1999 and 2019. After the exclusion of patients without oral cancer, oropharyngeal cancer, or hypopharyngeal cancer and patients with missing demographic information and information on behavioral factors, 2961 eligible patients (2788 male and 173 female) were included. The distribution of the clinicopathological characteristics of the HNC patients is shown in Table S1 of the Supplementary Materials. A total of 11,462 healthy controls (5650 male and 5812 female) were selected from the Taiwan Biobank (*n* = 122,071), according to the age- and sex-specific distribution of the general Taiwanese population in 2013 (Figure 1).

### 2.2. Data Collection

Data from the participants were collected in face-to-face interviews using a structured lifestyle questionnaire that included questions regarding sociodemographic characteristics (age, sex, education, parental ethnicity, and marital status), lifestyle habits (active and passive smoking, alcohol consumption, betel-nut chewing, and tea and coffee consumption), dietary habits, family history of oral cancer, and body mass index (BMI). Additionally, the clinicopathological data, including tumor differentiation, tumor stage, tumor size, treatment methods, and prognosis, were gathered from the medical records.

The variables measured in the present study were the following: age (<40, 40–49, 50–59, ≥60 years); sex (male or female); education (junior high and below, senior high school, or college and above); parental ethnicity (Taiwanese, Hakka, or other); occupation (non-white collar or white collar); family history of oral cancer (yes or no); BMI (<18.5, 18.5–23.9, ≥24); active smoking, passive smoking, betel-nut chewing, and alcohol consumption (never or ever); and tea consumption, coffee consumption, and a vegetarian diet (yes or no).

### 2.3. Statistical Analysis

The chi-square test was performed to compare the categorical demographic characteristics between the case and control groups. Univariate logistic regression was used to observe the relationship between risk factors and the risk of HNC and to estimate the odds ratios (ORs) with 95% confidence intervals (CIs). All variables with *p* < 0.05 in the univariate logistic analyses were further assessed using multivariable logistic regression and stepwise selection. Based on the results from the final multivariate logistic regression models, we generated nomograms to estimate the risk of HNC. Calibration curves were plotted to calibrate the HNC risk-prediction models. The C-index was used to evaluate the discriminative ability; 0.5 indicated that the model had no predictive effect, with 1 indicating that the model’s prediction results were consistent with the actual results. Additionally, the risk-prediction models were subjected to 100, 500, and 1000 bootstrap resamples for internal validation, to assess the predictive accuracy. All statistical analyses were conducted using SAS software, version 9.4 (SAS Institute, Cary, NC, USA). Statistical significance was considered at *p* < 0.05.

## 3. Results

In total, 2961 patients with HNC (2788 men and 173 women) and 11,462 controls (5650 men and 5812 women) were included in the study. Regarding the tumor subsites among male cases, 2440 were oral cavity tumors, 175 were oropharyngeal tumors, and 173 were hypopharyngeal tumors. The demographic and lifestyle habits of the cases and controls are listed in Table 1. Patients with HNC were primarily male, older, predominantly Hakka, and had a history of alcohol and tobacco use, passive smoking, betel-nut chewing, tea consumption, and a lower BMI compared with the controls (all *p* < 0.001). In the univariate analysis, being male, old age, married status, Hakka ethnicity, occupation (non-white collar), ever using alcohol and tobacco, passive smoking, ever chewing betel nuts, tea consumption, a family history of oral cancer, and having a BMI of <18.5 were associated with an increased risk of HNC. Higher education level, coffee consumption, and a BMI ≥ 24 were associated with a protective effect against HNC. In the multivariate model, being male (OR: 2.86, 95% CI: 2.25–3.64), aged ≥ 60 (OR: 1.45, 95% CI: 1.12–1.87), of Hakka ethnicity (OR: 2.21, 95% CI: 1.80–2.71), ever consuming alcohol (OR: 3.33, 95% CI: 2.85–3.89), ever smoking cigarettes (OR: 2.34, 95% CI: 1.91–2.85), passive smoking (OR: 5.81, 95% CI: 5.00–6.75), ever chewing betel nuts (OR: 6.83, 95% CI: 5.76–8.11), tea consumption (OR: 1.47, 95% CI: 1.27–1.70), and a BMI of <18.5 (OR: 4.95, 95% CI: 3.32–7.39) were significantly associated with an increased risk of HNC. However, a higher education level (OR: 0.04, 95% CI: 0.04–0.06), coffee consumption (OR: 0.65, 95% CI: 0.55–0.77), and a BMI ≥ 24 (OR: 0.57, 95% CI: 0.49–0.67) were significantly protective against HNC.

Table 2 presents the demographic characteristics and lifestyle habits associated with HNC risk among male patients. The distribution of demographic characteristics and lifestyle habits is similar to that in Table 1. In the univariate analysis, older age, marital status, Hakka ethnicity, occupation (non-white collar), ever consuming alcohol, ever smoking cigarettes, passive smoking, ever chewing betel nuts, tea consumption, a BMI of <18.5, and a family history of oral cancer were associated with an increased risk of HNC. A higher education level, coffee consumption, a vegetarian diet, and a BMI ≥ 24 were associated with a lower risk of HNC. In the multivariate model, an age of 40–49 years (OR: 1.46, 95% CI: 1.15–1.84), Hakka ethnicity (OR: 2.38, 95% CI: 1.90–2.97), ever consuming alcohol (OR: 3.32, 95% CI: 2.82–3.89), ever smoking cigarettes (OR: 2.52, 95% CI: 2.04–3.12), passive smoking (OR: 4.86, 95% CI: 4.13–5.70), ever chewing betel nuts (OR: 6.15, 95% CI: 5.17–7.32), tea consumption (OR: 1.49, 95% CI: 1.27–1.74), and a BMI of <18.5 (OR: 4.32, 95% CI: 2.60–7.18) were significantly associated with an increased risk of HNC. A higher education level (OR: 0.04–0.18), coffee consumption (OR: 0.67 95%, CI: 0.56–0.80), and a BMI ≥ 24 (OR: 0.53, 95% CI: 0.45–0.62) were significantly protective against HNC.

Table 3 presents the demographic characteristics and lifestyle habits associated with HNC risk among female patients. Marital status, the father’s ethnicity, tea consumption, a vegetarian diet, and a family history of oral cancer were similarly distributed between cases and controls (all *p* > 0.05). In the univariate analysis, older age, Hakka ethnicity, occupation (non-white collar), ever consuming alcohol, ever smoking cigarettes, passive smoking, ever chewing betel nuts, and a BMI of <18.5 were associated with an increased risk of HNC. In the multivariate model, age, ever consuming alcohol (OR: 5.31, 95% CI: 2.96–9.54), ever smoking cigarettes (OR: 2.73, 95% CI: 1.55–4.82), passive smoking (OR: 19.0, 95% CI: 12.8–28.3), and a BMI of <18.5 (OR: 9.46, 95% CI: 4.73–18.9) were significantly associated with an increased risk of HNC. A higher education level (OR: 0.05, 95% CI: 0.02–0.09), coffee consumption (OR: 0.61, 95% CI: 0.39–0.94) and a BMI ≥ 24 (OR: 0.83, 95% CI: 0.56–1.24) were significant protective factors against HNC.

Table 4 shows tumor site-specific multivariate models for the risk of HNC among male patients. Age, Hakka ethnicity, ever consuming alcohol, ever smoking cigarettes, passive smoking, ever chewing betel nuts, and a BMI of <18.5 significantly increased the risks for all HNC subsites. Additionally, alcohol consumption exhibited the strongest positive association with cancer in the hypopharynx (OR: 14.3), followed by cancers in the oropharynx (OR: 9.67) and oral cavity (OR: 2.98). Cigarette smoking presented the strongest positive association with tumor subsites in the hypopharynx (OR: 6.94), followed by tumor subsites in the oropharynx (OR: 3.35) and oral cavity (OR: 2.39). Betel-nut chewing had the strongest positive association with oral cavity cancer (OR: 6.34), followed by hypopharyngeal cancer (OR: 4.74) and oropharyngeal cancer (OR: 3.68). Education level and a BMI ≥ 24 had significant protective effects against all HNC subsites. Coffee consumption exhibited a specific protective factor against oral cancer (OR: 0.68) and hypopharyngeal cancer (OR: 0.28). However, tea consumption was a specific risk factor for oral cancer (OR: 1.53).

**Figure 2:**
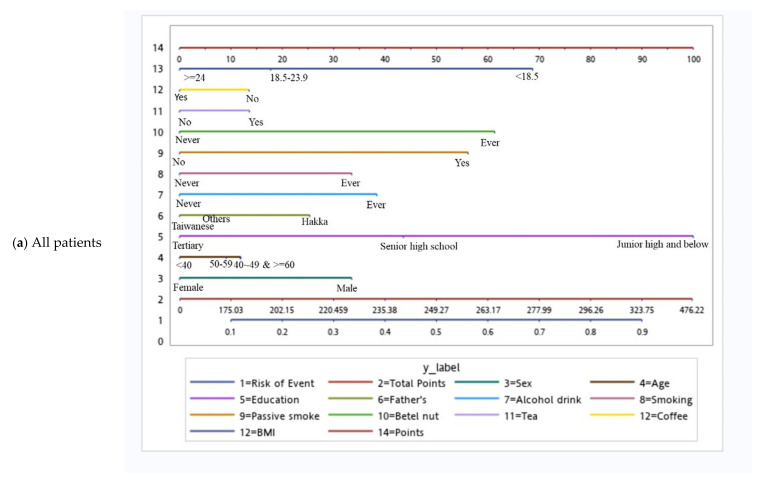

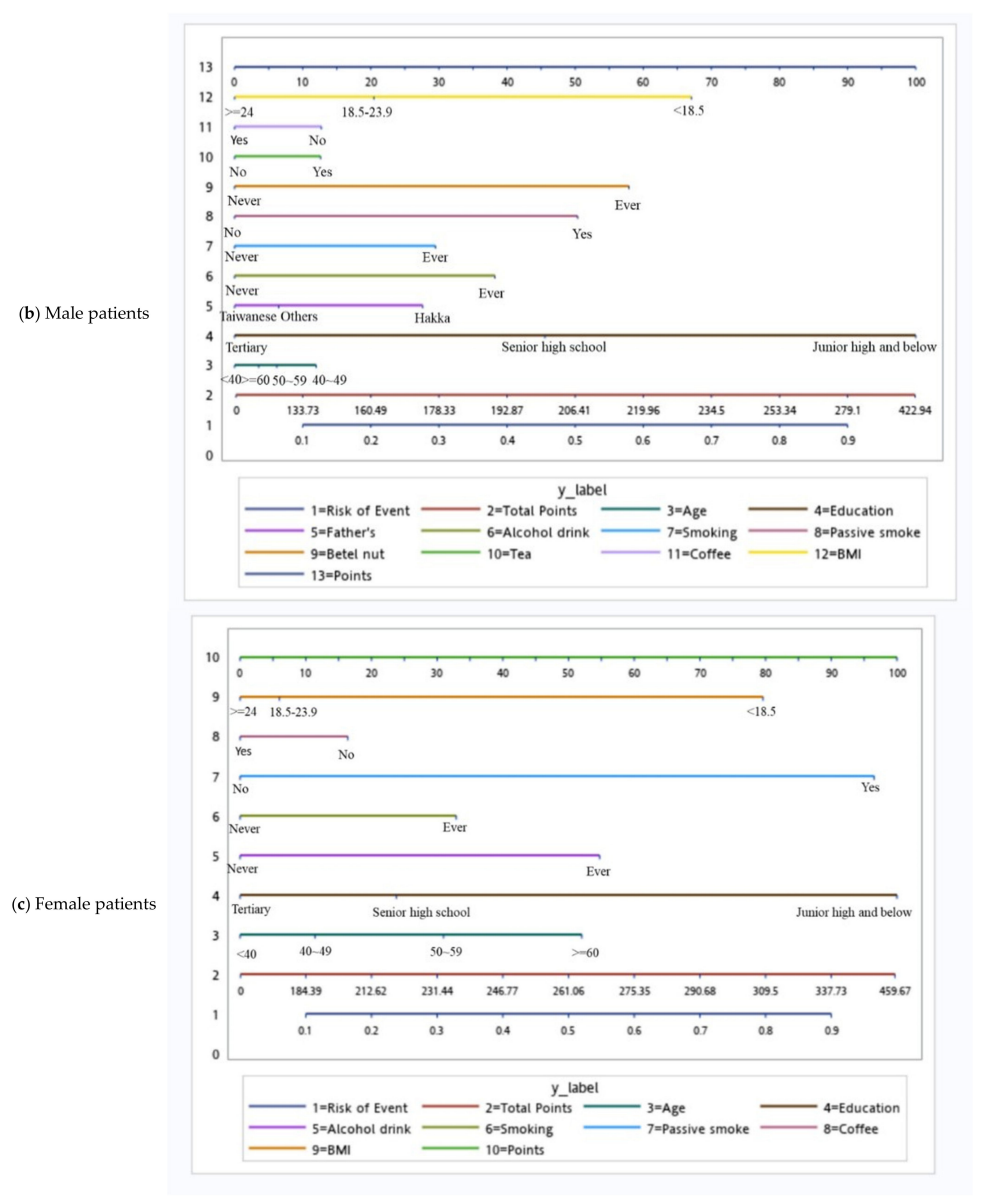
Nomograms of patients.

**Figure 3:**
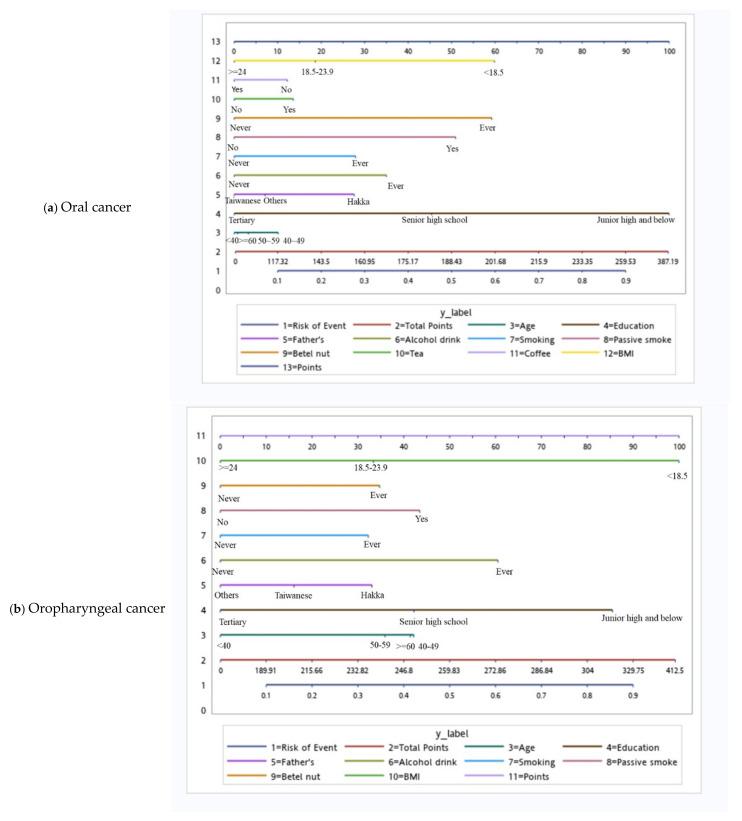

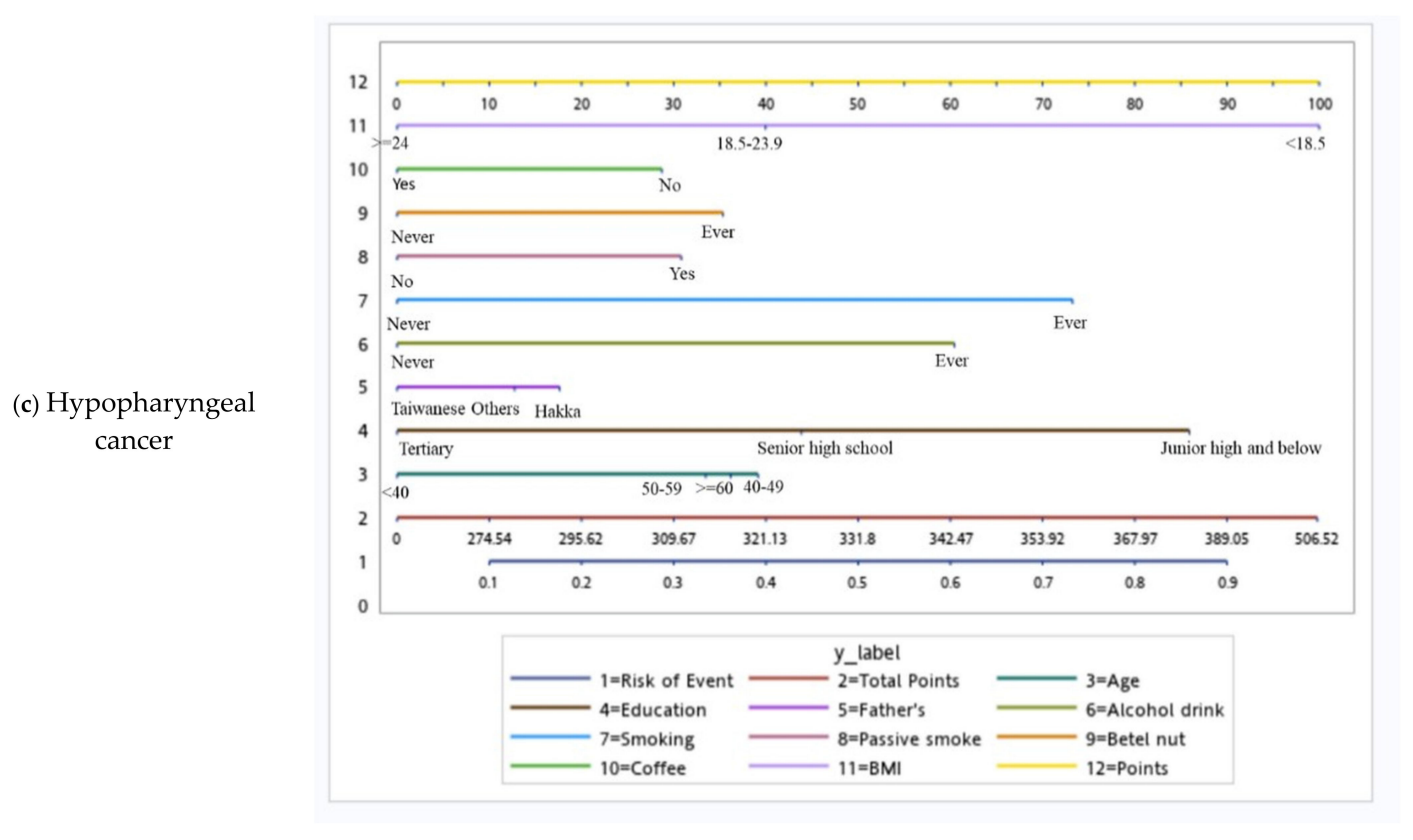
Tumor site-specific nomograms for male head and neck cancer.

Based on the independent risk factors for HNC, we plotted nomograms to predict the probability of developing HNC for all participants, for participants according to sex, and for participants with specific tumor subsites (Figure 2 and Figure 3). The score for each independent variable can be determined by drawing a vertical line to the point axis. The total risk score was then calculated by summing each variable score. Finally, the probability of HNC occurrence can be determined on the total point axis. Furthermore, the prediction models were internally validated using bootstrap resampling, which assessed the optimism-corrected discrimination and calibration (Table S2 in the Supplementary Materials). Across the 100, 500, and 1000 bootstrap repetitions, the optimism-corrected c-index for predicting the risk of HNC in all prediction models ranged from 0.93 to 0.98. The expected and observed risks were plotted for all prediction models, and all models had good calibration. The 45° line indicates perfect calibration (Figures S1–S6 in the Supplementary Materials).

## 4. Discussion

We established sex-specific and tumor subsite-specific prediction models of HNC risks for the general Taiwanese population. The HNC risk prediction models included sex, age, education level, father’s ethnicity, alcohol consumption, cigarette smoking, passive smoking, betel-nut chewing, tea consumption, coffee consumption, and BMI.

Among all sociodemographic factors, education level was the strongest predictor, which is consistent with other studies [13,14]. Previous analyses have demonstrated that lower education and income levels are associated with a higher risk of HNC. Among all lifestyle factors, alcohol consumption, cigarette smoking, and betel-nut chewing are major risk factors for HNC, and the effect of betel-nut chewing is greater than those of alcohol drinking or cigarette smoking, which is consistent with other studies in East Asia [4,15]. Betel-nut chewing is a key predictor in East Asian countries and increases the risk of oropharyngeal cancer, oral leukoplakia, oral mucosal lesions, and oral cancer [16,17]. Tobacco is a known human carcinogen [18]; one study suggested that secondhand smoke may increase the risk of breast cancer, nasal sinus cavity cancer, and nasopharyngeal cancer among adults [19]. Our research indicated that passive smoking is a risk factor for HNC, which is consistent with the literature [20]; however, the magnitude is higher in the present study than in other studies, which may be due to the exposure time and dose. Further studies are required to clarify the association between passive smoking and HNC.

Tea consumption is identified as a risk factor in our study, but a significant inverse association between tea consumption and HNC has been reported in five other studies [21,22,23,24,25]. Several factors may contribute to inconsistencies in the results. First, most of the studies were conducted in Europe and the United States, where tea consumption is not as prevalent as in Taiwan. Moreover, the types of tea that are consumed vary; for example, black tea is consumed the most in Europe and the United States. Second, the association between smoking and drinking tea is high in Asia [26]. This was also true in our study sample (the prevalence of tea consumption was 48.2% among patients who smoked and 32.6% among patients who did not smoke in the control group). Consequently, our results may be biased toward the null or overestimated. Consistent with the results of other studies [27], our analysis revealed that coffee consumption is protective against HNC. Our results also indicated that being underweight is significantly associated with a higher risk of HNC, while being overweight or obese is significantly associated with a lower risk of HNC. In another study that explored the association between BMI and 22 specific cancer sites, the results indicated that obesity has a protective effect against tumors in certain locations [28]; this phenomenon has been termed the “obesity paradox” [28]. A possible explanation for this phenomenon is that an increase in nutritional reserves and a higher body mass protect the body during periods of acute sickness.

Age, education level, alcohol consumption, cigarette smoking, passive smoking, coffee consumption, and BMI are common predictors of HNC among both men and women, and these predictors are consistent with other studies [29,30]. The father’s ethnicity, betel-nut chewing habits, and tea consumption are specific predictors of HNC among men. In particular, betel-nut chewing is much more prevalent among men over 18 years old than among women (22% vs. 1%) [31]. The risk of tea consumption between men and women is inconsistent with other studies [32,33]. Other studies may have evaluated participants who did not smoke or drink, so the confounding effects caused by these two factors can be excluded. Furthermore, the previous studies recruited more female participants, which may have affected the results.

In the prediction model for tumor subsites among men, the risk factors are the same as those for HNC. Coffee consumption is associated with the risk of oral cancer and hypopharyngeal cancer, which is consistent with other studies [27,34,35]. Additionally, the risks of alcohol consumption, cigarette smoking, and betel nut chewing vary based on the tumor sites. Alcohol consumption is associated with the greatest risk of hypopharyngeal cancer (OR: 14.32), followed by oropharyngeal (OR: 9.67) and oral cancer (OR: 2.98). Studies have reported that pharyngeal cancer is strongly associated with alcohol consumption [36]. In Taiwan, the incidence of hypopharyngeal cancer has risen the fastest at sites unrelated to human papillomavirus (HPV) [37], which may be explained by the high level of alcohol consumption among the Taiwanese population. We speculate that the reason why drinking has a greater effect on the hypopharynx may be related to the Taiwanese “bottoms-up” drinking tradition of ganbei [38] because it increases the exposure to alcohol in the hypopharynx, which subsequently increases the risk of lesions in the hypopharyngeal tissue [39,40,41]. Smoking posed the greatest risk of hypopharyngeal cancer (OR: 6.94), followed by oropharyngeal cancer (OR: 3.35) and oral cancer (OR: 2.39), which is consistent with other studies [42] and may be associated with differences in smoking patterns (e.g., inhalation). In a Japanese study on cigarette smoke inhalation and lung cancer risk, smokers who inhaled cigarette smoke had a 1.5-fold increase in risk compared with those who did not inhale cigarette smoke [43]. Unfortunately, evaluating smoking patterns is outside the scope of the present study. Finally, the risk of betel-nut chewing was the highest among patients with oral cancer (OR: 6.34), followed by hypopharyngeal (OR: 4.74) and oropharyngeal cancer (OR: 3.68). The risk of betel-nut chewing also depends on individual chewing habits. In Taiwan, betel nut is often held in the mouth for a long time before swallowing [44], which may increase the exposure time in the oral cavity.

The present study has several strengths. First, our study recruited a relatively large sample size, which drew upon patient records in the Taiwan Biobank. Second, we included more predictors (demographic factors, lifestyle factors) that could predict the risk of developing HNC in the general population more widely. Third, using nomograms could easily construct risk scores for each individual’s probability of the occurrence of HNC. However, this study has some limitations. First, few female patients with HNC were included in our study, which may have resulted in a type-2 error, an overestimation of the results, and a less stratified analysis of the tumor subsites. Second, the present study was a case-control study; therefore, recall bias may have occurred. Patients with HNC may have exaggerated their tobacco or alcohol use, compared with the controls. Third, selection bias may have also occurred. Linkou Chang Gung Memorial Hospital is close to several industrial parks in northern Taiwan; many individuals seeking medical services from this hospital are blue-collar laborers. Therefore, the patient cohort may include more men who are younger and have a lower socioeconomic status, which may have caused an overestimation of the effect of each predictor on HNC risk. However, these factors were adjusted for in the multivariate model. Fourth, although other risk factors, such as dietary habits, serological and genetic biomarkers, and HPV infection status were not collected, all established prediction models with c-indices of >0.9 indicated excellent discrimination in the analysis. Finally, although our prediction models were initially validated using bootstrap sampling methods, further research with larger multicenter cohorts is necessary to validate our findings.

## 5. Conclusions

For this study, we developed HNC risk prediction models for all patients, male patients, female patients, and male patients with specific tumor sites. The results indicate that the risk factors of HNC vary based on sex and tumor subsite. Our group-specific HNC risk prediction models may be used in clinical settings or communities to identify high-risk individuals, as well as to promote healthier behaviors, such as ceasing or reducing cigarette smoking, alcohol drinking, and betel-nut chewing. Such preventive efforts are the most effective way to reduce the occurrence of HNC in Taiwan. Furthermore, additional serological and genetic biomarkers should be included to increase the discriminatory ability of HNC risk prediction models.

## Figures and Tables

**Table 1:** Demographic characteristics and lifestyle associated with the risk of head and neck cancer.

	Case	(*n* = 2961)	Control	(*n* = 11,462)	Univariate		Multivariate	
Variable	*n*	%	*n*	%	OR (95% CI)	*p*-Value	OR (95% CI)	*p*-Value
Sex								
Male	2788	94.16	5650	49.29	16.6 (14.2–19.4)	<0.001	2.86 (2.25–3.64)	<0.001
Female	173	5.84	5812	50.71	1		1	
Age in years								
<40	387	13.07	3358	29.30	1		1	
40–49	956	32.29	3129	27.30	2.65 (2.33–3.01)	<0.001	1.45 (1.17–1.82)	0.001
50–59	944	31.88	3009	26.25	2.72 (2.40–3.09)	<0.001	1.33 (1.06–1.67)	0.014
≥60	674	22.76	1966	17.15	2.98 (2.59–3.41)	<0.001	1.45 (1.12–1.87)	0.004
Education level								
Junior high and below	1990	67.21	1505	13.13	1		1	
Senior high school	789	26.65	3341	29.15	0.18 (0.16–0.20)	<0.001	0.17 (0.14–0.20)	<0.001
Tertiary	182	6.15	6616	57.72	0.02 (0.02–0.02)	<0.001	0.04 (0.04–0.06)	<0.001
Marital status								
Unmarried	281	9.49	1478	12.89	1		NS	
Married	2680	90.51	9984	87.11	1.41 (1.23–1.62)	<0.001		
Mother’s ethnicity								
Taiwanese	2251	76.02	9450	82.45	1		NS	
Hakka	569	19.22	1227	10.70	1.95 (1.75–2.17)	<0.001		
Others	141	4.76	785	6.85	0.76 (0.63–0.91)	0.003		
Father’s ethnicity								
Taiwanese	2184	73.76	8685	75.77	1		1	
Hakka	559	18.88	1144	9.98	1.94 (1.74–2.17)	<0.001	2.21 (1.80–2.71)	<0.001
Others	218	7.36	1633	14.25	0.53 (0.46–0.62)	<0.001	1.20 (0.94–1.54)	0.145
Occupation								
White collar	908	30.67	5628	49.10	1		NS	
Non-white collar	2053	69.33	5834	50.90	2.18 (2.00–2.38)	<0.001		
Alcohol-drinking								
Never	967	32.66	10,300	89.96	1		1	
Ever	1994	67.34	11,162	10.14	18.3 (16.6–20.2)	<0.001	3.33 (2.85–3.89)	<0.001
Cigarette smoking								
Never	364	12.29	8732	76.18	1		1	
Ever	2597	87.71	2730	23.82	22.8 (20.3–25.7)	<0.001	2.34 (1.91–2.85)	<0.001
Passive smoking								
No	1113	37.59	9903	86.40	1		1	
Yes	1848	62.41	1559	13.60	10.6 (9.62–11.6)	<0.001	5.81 (5.00–6.75)	<0.001
Betel-nut chewing								
Never	564	19.05	10523	91.81	1		1	
Ever	2397	80.95	939	8.19	47.6 (42.5–53.4)	<0.001	6.83 (5.76–8.11)	<0.001
Tea drinking								
No	1462	49.38	7296	63.65	1		1	
Yes	1499	50.62	4166	36.35	1.80 (1.66–1.95)	<0.001	1.47 (1.27–1.70)	<0.001
Coffee drinking								
No	2380	80.38	7332	63.97	1		1	
Yes	581	19.62	4130	36.03	0.43 (0.39–0.48)	<0.001	0.65 (0.55–0.77)	<0.001
Vegetarian								
No	2816	95.10	10,397	90.71	1		NS	
Yes	145	4.90	1065	9.29	0.50 (0.42–0.60)	<0.001		
BMI in kg/m^2^								
<18.5	192	6.48	302	2.63	2.54 (2.10–3.08)	<0.001	4.95 (3.32–7.39)	<0.001
18.5–23.9	1348	45.53	5393	47.05	1		1	
≥24	1421	47.99	5767	50.31	0.99 (0.91–1.07)	0.736	0.57 (0.49–0.67)	<0.001
Family history of oral cancer								
No	2907	98.18	11,316	98.73	1		NS	
Yes	54	1.82	146	1.27	1.44 (1.05–1.97)	<0.001		

NS: non-selected.

**Table 2:** Demographic characteristics and lifestyle associated with the risk of head and neck cancer among male patients.

	Case	(*n* = 2788)	Control	(*n* = 5650)	Univariate		Multivariate	
Variable	*n*	%	*n*	%	OR (95% CI)	*p*-Value	OR (95% CI)	*p*-Value
Age in years								
<40	371	13.31	1667	29.50	1		1	
40–49	924	33.14	1554	27.50	2.67 (2.33–3.07)	<0.001	1.46 (1.15–1.84)	0.002
50–59	893	32.03	1486	26.30	2.70 (2.35–3.11)	<0.001	1.22 (0.95–1.55)	0.115
≥60	600	21.52	943	16.69	2.86 (2.46–3.33)	<0.001	1.12 (0.85–1.47)	0.428
Education level								
Junior high and below	1856	66.57	559	9.89	1		1	
Senior high school	762	27.33	1468	25.98	0.16 (0.14–0.18)	<0.001	0.18 (0.15–0.22)	<0.001
Tertiary	170	6.10	3623	64.12	0.01 (0.01–0.02)	<0.001	0.04 (0.03–0.06)	<0.001
Marital status								
Unmarried	265	9.51	711	12.58	1		NS	
Married	2523	90.49	4939	87.42	1.37 (1.18–1.59)	<0.001		
Mother’s ethnicity								
Taiwanese	2124	76.18	4715	83.45	1		NS	
Hakka	540	19.37	592	10.48	2.03 (1.78–2.30)	<0.001		
Others	124	4.45	343	6.07	0.80 (0.65–0.99)	0.042		
Father’s ethnicity								
Taiwanese	2059	73.85	4297	76.05	1		1	
Hakka	533	19.12	564	9.98	1.97 (1.73–2.25)	<0.001	2.38 (1.90–2.97)	<0.001
Others	196	7.03	789	13.96	0.52 (0.44–0.61)	<0.001	1.23 (0.94–1.60)	0.140
Occupation							NS	
White collar	844	30.27	2509	44.41	1			
Non-white collar	1944	69.73	3141	55.59	1.84 (1.67–2.03)	<0.001		
Alcohol drinking								
Never	835	29.95	4623	81.82	1		1	
Ever	1953	70.05	1027	18.18	10.5 (9.47–11.7)	<0.001	3.32 (2.82–3.89)	<0.001
Cigarette smoking								
Never	233	8.36	3200	56.64	1		1	
Ever	2555	91.64	2450	43.36	14.3 (12.4–16.5)	<0.001	2.52 (2.04–3.12)	<0.001
Passive smoking								
No	1059	37.98	4731	83.73	1		1	
Yes	1729	62.02	919	16.27	8.41 (7.57–9.33)	<0.001	4.86 (4.13–5.70)	<0.001
Betel-nut chewing								
Never	434	15.57	4725	83.63	1		1	
Ever	2354	84.43	925	16.37	27.7 (24.5–31.4)	<0.001	6.15 (5.17–7.32)	<0.001
Tea drinking								
No	1353	48.53	3252	57.56	1		1	
Yes	1435	51.47	2398	42.44	1.44 (1.31–1.58)	<0.001	1.49 (1.27–1.74)	<0.001
Coffee drinking								
No	2246	80.56	3726	65.95	1		1	
Yes	542	19.44	1924	34.05	0.47 (0.42–0.52)	<0.001	0.67 (0.56–0.80)	<0.001
Vegetarian								
No	2668	95.70	5200	92.04	1		NS	
Yes	120	4.30	450	7.96	0.52 (0.42–0.64)	<0.001		
BMI in kg/m^2^								
<18.5	170	6.10	66	1.17	4.15 (3.10–5.56)	<0.001	4.32 (2.60–7.18)	<0.001
18.5–23.9	1273	45.66	2051	36.30	1		1	
≥24	1345	48.24	3533	62.53	0.61 (0.56–0.67)	<0.001	0.53 (0.45–0.62)	<0.001
Family history of oral cancer								
No	2737	98.17	5582	98.80	1		NS	
Yes	51	1.83	68	1.20	1.53 (1.06–2.21)	0.023		

NS: non-selected.

**Table 3:** Demographic characteristics and lifestyle associated with the risk of head and neck cancer among female patients.

	Case	(*n* = 173)	Control	(*n* = 5812)	Univariate		Multivariate	
Variable	*n*	%	*n*	%	OR (95% CI)	*p*-Value	OR (95% CI)	*p*-Value
Age in years								
<40	16	9.25	1691	29.09	1		1	
40–49	32	18.50	1575	27.10	2.15 (1.17–3.93)	0.013	1.42 (0.69–2.93)	0.347
50–59	51	29.48	1523	26.20	3.54 (2.01–6.23)	<0.001	2.57 (1.25–5.28)	0.01
≥60	74	42.77	1023	17.60	7.65 (4.43–13.2)	<0.001	4.89 (2.36–10.2)	<0.001
Education level								
Junior high and below	134	77.46	946	16.28	1		1	
Senior high school	27	15.61	1873	32.33	0.10 (0.07–0.16)	<0.001	0.10 (0.06–0.16)	<0.001
Tertiary	12	6.94	2993	51.50	0.03 (0.02–0.05)	<0.001	0.05 (0.02–0.09)	<0.001
Marital status								
Unmarried	16	9.25	767	13.20	1		NS	
Married	157	90.75	5045	86.80	1.49 (0.89–2.51)	0.132		
Mother’s ethnicity								
Taiwanese	127	73.41	4735	81.47	1		NS	
Hakka	29	16.76	635	10.93	1.70 (1.13–2.57)	0.011		
Other	17	9.83	442	7.60	1.43 (0.86–2.40)	0.171		
Father’s ethnicity								
Taiwanese	125	72.25	4388	75.50	1		NS	
Hakka	26	15.03	580	9.98	1.57 (1.02–2.42)	0.039		
Other	22	12.72	844	14.52	0.92 (0.58–1.45)	0.705		
Occupation								
White collar	64	36.99	3119	53.66	1		NS	
Non-white collar	109	63.01	2693	46.34	1.97 (1.44–2.70)	<0.001		
Alcohol drinking								
Never	132	76.30	5677	97.68	1		1	
Ever	41	23.70	135	2.32	13.1 (8.85–19.3)	<0.001	5.31 (2.96–9.54)	<0.001
Cigarette smoking								
Never	131	75.72	5532	95.18	1		1	
Ever	42	24.28	280	4.82	6.34 (4.39–9.15)	<0.001	2.73 (1.55–4.82)	0.001
Passive smoking								
No	54	31.21	5172	88.99	1		1	
Yes	119	68.79	640	11.01	17.8 (12.8–24.8)	<0.001	19.0 (12.8–28.3)	<0.001
Betel-nut chewing								
Never	130	75.14	5798	99.76	1		NS	
Ever	43	24.86	14	0.24	137.0 (3.13–256.7)	<0.001		
Tea drinking								
No	109	63.01	4044	69.58	1		NS	
Yes	64	36.99	1768	30.42	1.34 (0.98–1.84)	0.065		
Coffee drinking								
No	134	77.46	3606	62.04	1		1	
Yes	39	22.54	2206	37.96	0.48 (0.33–0.68)	<0.001	0.61 (0.39–0.94)	0.025
Vegetarian								
No	148	85.55	5197	89.42	1		NS	
Yes	25	14.45	615	10.58	1.43 (0.93–2.20)	0.106		
BMI in kg/m^2^								
<18.5	22	12.72	236	4.06	4.15 (2.54–6.80)	<0.001	9.46 (4.73–18.9)	<0.001
18.5–23.9	75	43.35	3342	57.50	1		1	
≥24	76	43.93	2234	38.44	1.52 (1.10–2.10)	0.012	0.83 (0.56–1.24)	0.367
Family history of oral cancer								
No	170	98.27	5734	98.66	1		NS	
Yes	3	1.73	78	1.34	1.30 (0.41–4.15)	0.660		

NS: non-selected.

**Table 4:** Tumor site-specific multivariable models for the risk of head and neck cancer among male participants.

	Control	Oral			Oropharyngeal			Hypopharyngeal		
Variable	*n* = 5650	*n* = 2440	OR (95% CI)	*p*-Value	*n* = 175	OR (95% CI)	*p*-Value	*n* = 173	OR (95% CI)	*p*-Value
Age in years										
<40	1667	347	1		11	1		13	1	
40–49	1554	803	1.37 (1.08–1.74)	0.001	63	4.86 (2.21–10.7)	<0.001	58	5.61 (2.34–13.4)	<0.001
50–59	1486	774	1.11 (0.87–1.41)	0.418	60	3.84 (1.73–8.56)	0.001	59	4.36 (1.81–10.5)	0.001
≥60	943	516	1.03 (0.78–1.36)	0.859	41	4.73 (2.03–11.0)	<0.001	43	4.92 (1.94–12.5)	0.001
Education level										
Junior high and below	559	1615	1		114	1		127	1	
Senior high school	1468	669	0.18 (0.15–0.22)	<0.001	52	0.20 (0.13–0.31)	<0.001	41	0.16 (0.10–0.26)	<0.001
Tertiary	3623	156	0.04 (0.04–0.06)	<0.001	9	0.04 (0.02–0.09)	<0001	5	0.02 (0.01–0.06)	<0.001
Father’s ethnicity										
Taiwanese	4297	1803	1		136	1		120	1	
Hakka	564	468	2.36 (1.88–2.97)	<0.001	31	1.89 (1.09–3.27)	0.023	34	2.17 (1.18–4.00)	0.013
Others	789	169	1.25 (0.95–1.65)	0.115	8	0.55 (0.23–1.31)	0.177	19	1.75 (0.86–3.56)	0.122
Alcohol drinking										
Never	4623	796	1		23	1		16	1	
Ever	1027	1644	2.98 (2.53–3.51)	<0.001	152	9.67 (5.83–16.0)	<0.001	157	14.3 (7.68–26.7)	<0.001
Cigarette smoking										
Never	3200	219	1		10	1		4	1	
Ever	2450	2221	2.39 (1.92–2.97)	<0.001	165	3.35 (1.59–7.05)	0.001	169	6.94 (2.29–21.0)	0.001
Passive smoking										
No	4731	919	1		67	1		73	1	
Yes	919	1521	4.89 (4.15–5.76)	<0.001	108	5.10 (3.39–7.69)	<0.001	100	3.89 (2.48–6.09)	<0.001
Betel-nut chewing										
Never	4725	374	1		35	1		25	1	
Ever	925	2066	6.34 (5.30–7.59)	<0.001	140	3.68 (2.28–5.93)	<0.001	148	4.74 (2.73–8.22)	<0.001
Tea drinking										
No	3252	1156	1		100	NS		97	NS	
Yes	2398	1284	1.53 (1.30–1.80)	<0.001	75			76		
Coffee drinking										
No	3726	1947	1		145	NS		154	1	
Yes	1924	493	0.68 (0.57–0.82)	<0.001	30			19	0.28 (0.15–0.54)	<0.001
BMI in kg/m^2^										
<18.5	66	117	3.62 (2.10–6.22)	<0.001	25	12.2 (5.03–29.4)	<0.001	28	14.1 (5.07–39.0)	<0.001
18.5–23.9	2051	1090	1		86	1		97	1	
≥24	3533	1233	0.56 (0.47–0.66)	<0.001	64	0.29 (0.19–0.44)	<0.001	48	0.17 (0.11–0.28)	<0.001

NS: non-selected.

## Data Availability

The data presented in this study are available in this article (and Supplementary Materials).

## Supplementary Materials

The following supporting information can be downloaded at https://www.mdpi.com/article/10.3390/cancers14215338/s1, Table S1: Clinicopathological characteristics of head and neck cancer patients; Table S2: Original c-index and optimism, adjusting performance for the risk prediction model; Figure S1: Calibration plot for the head and neck cancer prediction model; Figure S2: Calibration plot for the men’s head and neck cancer prediction model; Figure S3: Calibration plot for the women’s head and neck cancer prediction model; Figure S4: Calibration plot for the oral cancer prediction model; Figure S5: Calibration plot for the oropharyngeal cancer prediction model; Figure S6: Calibration plot for the hypopharyngeal cancer prediction model.
