# Supplementary material for: Risk Prediction Models for Patients with Head and Neck Cancer among the Taiwanese Population
Source: Cancers (Basel). 2022 Oct 29;14(21):5338. doi: 10.3390/cancers14215338 (PMC9657226; doi:10.3390/cancers14215338)
Supplement: Supplementary file 1 [file cancers-14-05338-s001.zip › cancers-1947684-supplementary.pdf]

Table S1 Clinicopathological characteristics of head and neck cancer patients

Characteristic	All		Oral cavity		Oropharyngeal		Hypopharyngeal		*P* value
	N	%	N	%	N	%	N	%	
Pathologic t status									<0.001
T1	612	20.67	576	22.17	25	13.51	11	6.18	
T2	867	29.28	799	30.75	47	25.41	21	11.80	
T3	295	9.96	241	9.28	26	14.05	28	15.73	
T4	841	28.40	702	27.02	57	30.81	82	46.07	
Unknown	346	11.69	280	10.78	30	16.22	36	20.22	
Pathologic n status									<0.001
No	1572	53.09	1481	57.01	52	28.11	39	21.91	
N1	343	11.58	295	11.35	26	14.05	22	12.36	
N2	649	21.92	518	19.94	64	34.59	67	37.64	
N3	31	1.05	7	0.27	11	5.95	13	7.30	
Unknown	366	12.36	297	11.43	32	17.30	37	20.79	
Pathologic stage									<0.001
I	506	17.09	489	18.82	14	7.57	3	1.69	
II	524	17.70	501	19.28	19	10.27	4	2.25	
III	382	12.90	338	13.01	24	12.97	20	11.24	
IV	1203	40.63	990	38.11	98	52.97	115	64.61	
Unknown	346	11.69	280	10.78	30	16.22	36	20.22	
Differentiation									<0.001
Well	850	28.71	823	31.68	18	9.73	9	5.06	
Moderate	1383	46.71	1175	45.23	107	57.84	101	56.74	
Poor	254	8.58	199	7.66	23	12.43	32	17.98	
Unknown	474	16.01	401	15.43	37	20.00	36	20.22	

Table S2 Original c-index, optimism, adjust performance for risk prediction model

	Original c-index	Optimism	Adjust performance
All			
bootstrapping=100	0.9686 (0.9656-0.9716)	0.00048	0.96812
bootstrapping=500	0.9686 (0.9656-0.9716)	0.00048	0.96812
bootstrapping=1000	0.9686 (0.9656-0.9716)	0.00042	0.96818
Male			
bootstrapping=100	0.9558 (0.9517-0.9600)	0.00018	0.95562
bootstrapping=500	0.9558 (0.9517-0.9600)	0.00050	0.95530
bootstrapping=1000	0.9558 (0.9517-0.9600)	0.00060	0.95520
Female			
bootstrapping=100	0.9404 (0.9222-0.9587)	0.00181	0.93859
bootstrapping=500	0.9404 (0.9222-0.9587)	0.00321	0.93719
bootstrapping=1000	0.9404 (0.9222-0.9587)	0.00337	0.93703
Oral			
bootstrapping=100	0.9535 (0.9491-0.9580)	0.00035	0.95315
bootstrapping=500	0.9535 (0.9491-0.9580)	0.00047	0.95303
bootstrapping=1000	0.9535 (0.9491-0.9580)	0.00054	0.95296
Oropharyngeal			
bootstrapping=100	0.9724 (0.9652-0.9796)	0.00192	0.97048
bootstrapping=500	0.9724 (0.9652-0.9796)	0.00231	0.97009
bootstrapping=1000	0.9724 (0.9652-0.9796)	0.00233	0.97007
Hypopharyngeal			
bootstrapping=100	0.9819 (0.9754-0.9885)	0.00163	0.98027
bootstrapping=500	0.9819 (0.9754-0.9885)	0.00211	0.97979
bootstrapping=1000	0.9819 (0.9754-0.9885)	0.00217	0.97973

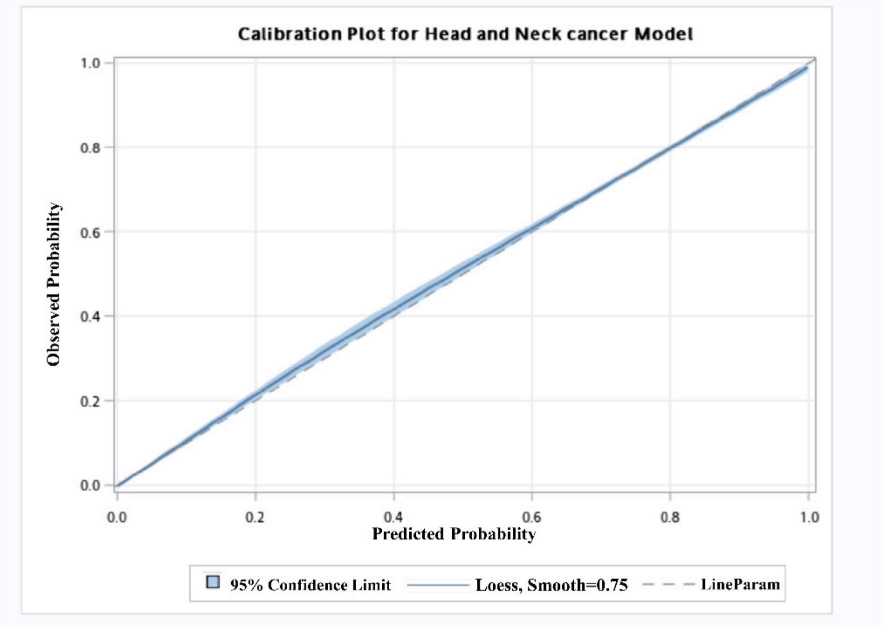

Figure S1 Calibration plot for head and neck cancer prediction model

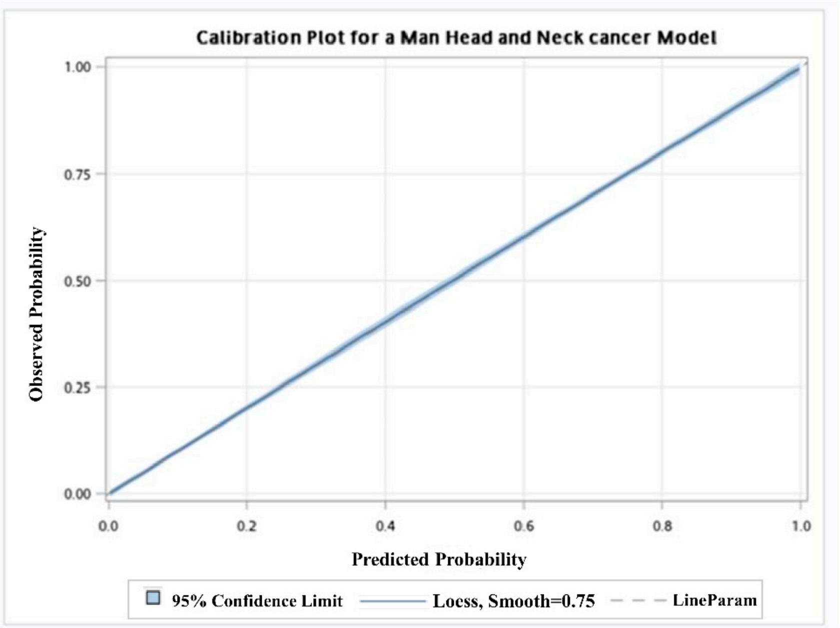

Figure S2 Calibration plot for men head and neck cancer prediction model

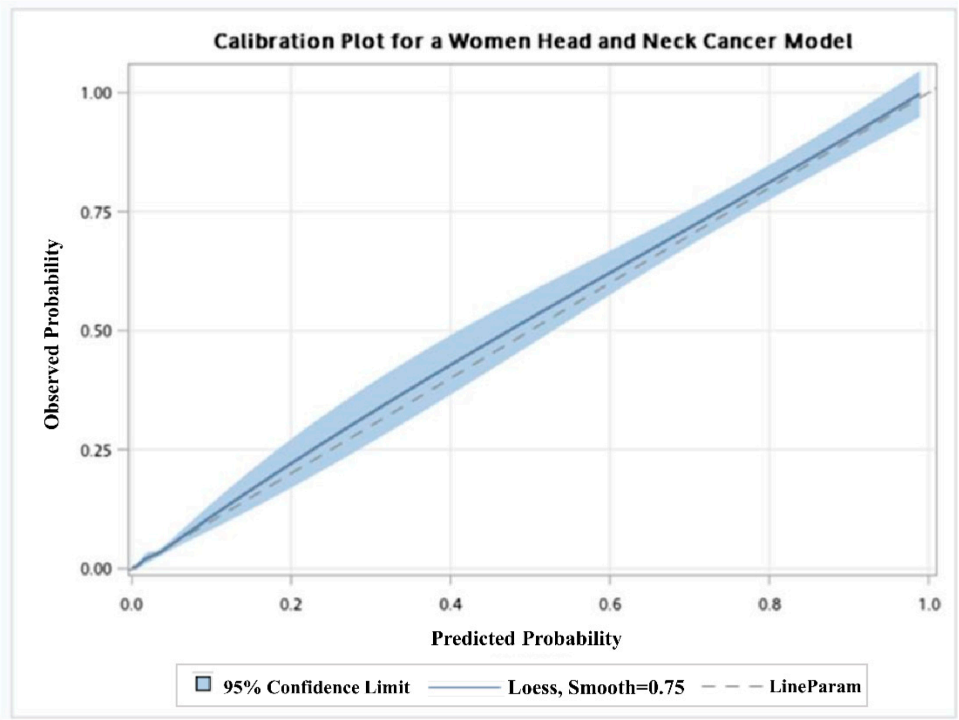

Figure S3 Calibration plot for women head and neck cancer prediction model

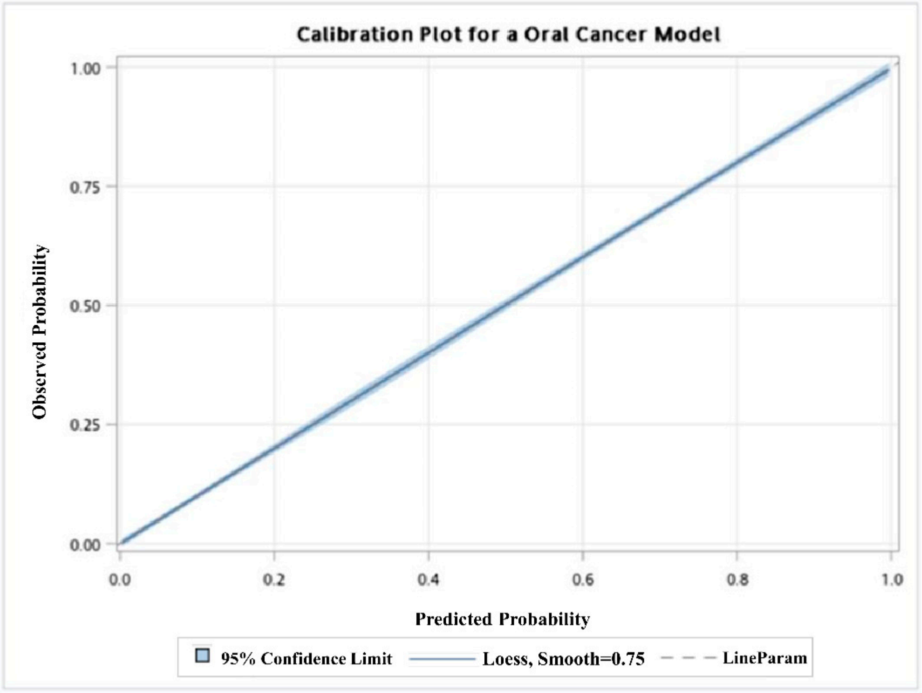

Figure S4 Calibration plot for oral cancer prediction model

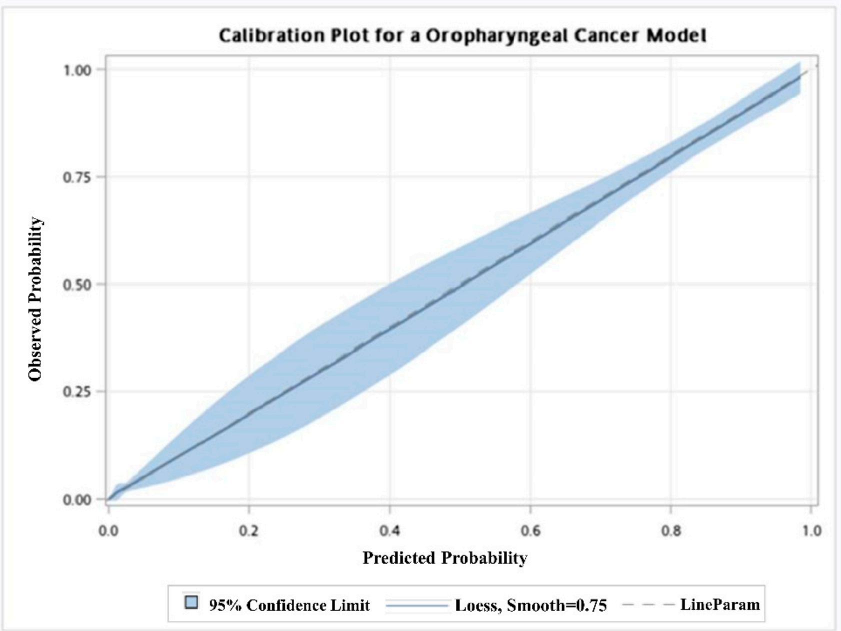

Figure S5 Calibration plot for oropharyngeal cancer prediction model

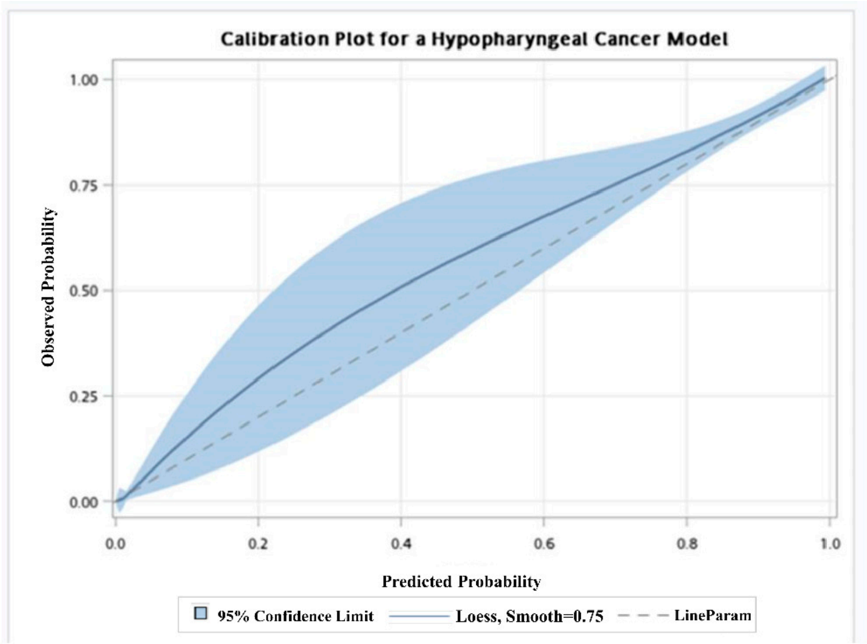

Figure S6 Calibration plot for hypopharyngeal cancer prediction model
